# The clinical value of the Tei index among Nigerians with hypertensive heart failure: correlation with other conventional indices

**DOI:** 10.5830/CVJA-2011-032

**Published:** 2012-02

**Authors:** AA Akintunde

**Affiliations:** Division of Cardiology, LAUTECH Teaching Hospital, Osogbo, Osun State, Nigeria; Cardiology Clinic, University Hospital of Tübingen, Department of Internal Medicine III, Eberhard Karls University, Tubingen,Germany

**Keywords:** hypertensive heart failure, correlation, Tei index, systolic dysfunction, diastolic dysfuntion, Africans, Doppler echocardiography

## Abstract

**Background:**

Various conventional methods are used for functional evaluation and risk stratification in heart failure. A combined index of global myocardial performance called the Tei index has been described. The aim of this study was to evaluate the correlation of the Tei index with other conventional indices of systolic and diastolic function among Nigerians with hypertensive heart failure.

**Methods:**

Fifty-five subjects with hypertensive heart failure and 30 controls were examined, a clinical history was taken, and echocardiography was performed on them. The subjects were categorised into four groups based on their ejection fraction (normal ejection fraction, mild, moderate and severe heart failure). The Tei index was calculated as the sum of the isovolumic relaxation and contraction time, divided by the ejection time. Statistical analysis was done using SPSS 16.0.

**Results:**

The Tei index was significantly higher among subjects with hypertensive heart failure compared with the controls (0.91 ± 0.33 vs 0.28 ± 0.16, *p* < 0.005). The Tei index also increased with the severity of the heart failure and was inversely correlated with ejection fraction (*r* = –0.697, *p* < 0.001) and fractional shortening (*r* = –0.580, *p* = 0.001). It was directly correlated with mitral E/A ratio (*r* = 0.246, *p* = 0.030), left ventricular internal diastolic dimension (*r* = 0.414, *p* = 0.002), left ventricular internal systolic dimension (*r* = 0.596, *p* < 0.001) and deceleration time (*r* = 0.219, *p* = 0.032).

**Conclusion:**

The Tei index correlated significantly with other conventional indices of systolic and diastolic function among Nigerians with hypertensive heart failure. It can be used as a risk-stratification index similar to other traditional indices of systolic and diastolic function.

## Abstract

Heart failure worldwide is increasingly becoming a serious public health concern as the population continually ages and the risk factors for heart failure are increasingly prevalent.^1^ Hypertension remains a common cause of heart failure in Africa.^2^ Congestive heart failure includes systolic and/or diastolic dysfunction; systolic dysfunction involves abnormalities with contractility, diastolic dysfunction is associated with relaxation abnormalities.

Heart failure is associated with significant morbidity and mortality.^1^ Long-term prognoses are comparable with malignancies.^2^ It is a progressive disease associated with significant economic losses.^3^ While systolic dysfunction predominates in some patients, others have diastolic dysfunction with normal ejection fraction. It has been suggested that heart failure subjects often have both systolic and diastolic function together, although the nomenclature is mostly based on the predominant type of dysfunction. An important modality in its management is risk stratification at diagnosis and during therapy.

Currently, echocardiography is the single most useful non-invasive investigation used in the risk stratification of subjects with heart failure.^4^ Echocardiographic parameters used in assessing systolic function include ejection fraction, cardiac output and fractional shortening, while diastolic function can be evaluated by transmitral Doppler inflow or pulmonary venous flow studies.^5^5,^6^ These have been closely related to prognosis.^7^ Each of these parameters is an independent but limited predictor of morbidity in subjects with heart failure. Therefore, a combined index of systolic and diastolic dysfunction may be a better prognostic index.

The Tei index was introduced in 1995.^8^ It is a combined estimate of the systolic and diastolic function obtained from Doppler echocardiography. Isolated assessment of systolic or diastolic dysfunction may not reflect the combined abnormality in such patients, and a combined myocardial performance index may be more effective in analysing the overall function/dysfunction in such patients.

The Tei index has been shown to correlate with other indices of systolic and diastolic function in subjects with heart failure, dilated cardiomyopathy, amyloidosis and congenital heart disease in other populations.^9^- ^12^ However, reports among blacks Africans are scarce. This study was set to evaluate the clinical value of the Tei index of overall myocardial performance among black African subjects with a clinical diagnosis of heart failure.

## Methods

Fifty-five consecutive subjects with a clinical diagnosis of hypertensive heart failure were included in this study. Thirty age- and gender-matched controls with a similar mean age were recruited as controls. They were patients’ relatives and hospital staff who gave their consent to participate in the study.

Congestive heart failure was clinically diagnosed by the presence of related symptoms of left and/or right heart failure according to the Framingham criteria. Hypertensive heart failure was diagnosed by ascertaining a previous diagnosis and treatment with antihypertensive therapy and the presence of peripheral stigmata of hypertension, such as locomotor brachialis, thickened arterial wall, cardiomegaly and loud aortic component of the second heart sound. All of these patients were on combination therapy including medications such as diuretics, angiotensin converting enzyme (ACE) inhibitors, angiotensin receptor blockers (ARBs), aspirin or warfarin.

Patients with diabetes, nephrotic syndrome, renal failure, present or past history of liver disease, and stroke were excluded from the study. Patients with echocardiographic evidence of valvular heart disease, dilated cardiomyopathy and restrictive cardiomyopathy as well as those with incomplete echocardiographic examinations and poor image windows were also excluded.

All subjects were examined and a clinical history was taken. Two dimensional (2D), M-mode and Doppler echocardiography were done in the left lateral decubitus position using a 3.5-MHz probe, according to standard recommendations of the American Society of Echocardiography.^13^ The 2D-derived M-mode was used to estimate the left ventricular wall and chamber dimensions and assess the ejection fraction and fractional shortening, using the Teichholz formula.^14^ The apical four- and five-chamber views were used to estimate the transmitral early (E), late atrial (A) and E/A velocities, deceleration time and isovolumic relaxation time. The isovolumic contraction time was derived from the Doppler study and taken as the time interval from the end of the mitral A wave and the beginning of the ejection time. The ejection time was obtained by Doppler echocardiography and was the time interval from the beginning to the end of the left ventricular outflow.

**Fig. 1:**
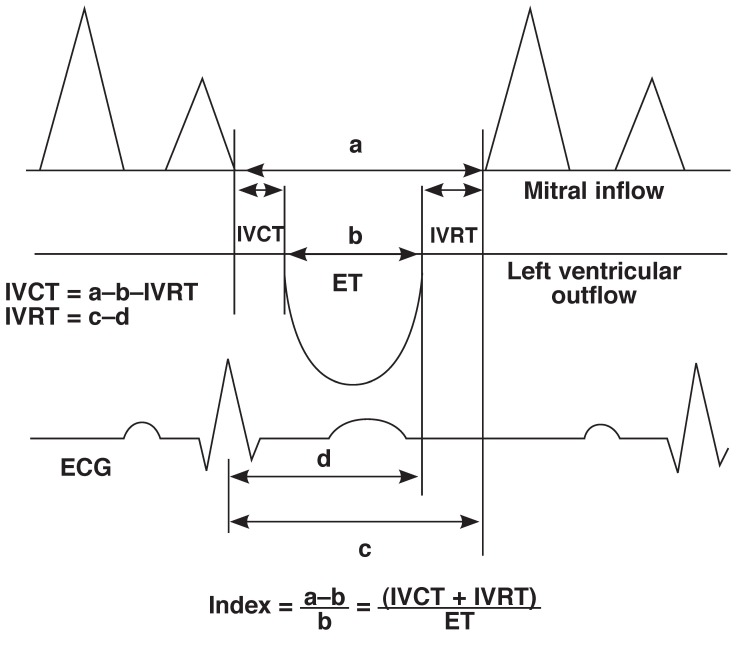
Schematic diagram of the method of estimating the components of the Tei index. IVCT: isovolumic contraction time, IVRT: isovolumic relaxation time, ET: ejection time.

In our laboratory, the intra-observer concordance correlation coefficient ranged from 0.80 to 0.96 while that of the inter-observer concordance ranged from 0.79 to 0.97. Hypertensive heart failure subjects were categorised, based on the ejection fraction, into normal ejection fraction, mild heart failure (ejection fraction 45–55%), moderate heart failure (ejection fraction 35–45%) and severe heart failure (ejection fraction < 35%). The Tei index was defined as the sum of the isovolumic relaxation time and isovolumic contraction time, divided by the ejection time obtained from the left ventricular inflow and outflow, as shown in Fig. 1.^15^

Ethical approval was obtained for the study. Statistical analysis was done using the Statistical package for Social Sciences 16. 0 (Chicago Ill). Data were summarised as means ± standard deviation (quantitative) and proportions and percentages (qualitative data). Comparison between groups was done by the independent *t*-test and chi-square test as appropriate. Statistical relationships between Doppler echocardiography measurements, Tei index and echocardiography-derived variables were done by correlation analysis. A *p*-value < 0.05 was taken as statistically significant.

## Results

The mean age of the subjects with hypertensive heart failure was 57.8 ± 14.2 years (age range 42–87 years) and comprised 26 females (47.3%), compared to 56.4 ± 18.6 years, with 19 females (47.5% females) for the control group. They were well matched in age distribution and gender. Hypertensive heart failure patients were receiving combination therapy including at least diuretics, ACE inhibitors and aspirin/warfarin.

Table 1 shows the clinical characteristics of the study participants. Ejection fraction and fractional shortening were lower among subjects with hypertensive heart failure compared to control subjects (48.5 ± 25.9, 22.5 ± 11.4 vs 70.6 ± 12.2, 38.7 ± 8.1, *p* < 0.005, respectively). Posterior wall thickness, inter-ventricular septal thickness, left ventricular internal dimension in diastole and systole, and left atrial dimensions were higher among subjects with hypertensive heart failure than control subjects. The Tei index was significantly higher among hypertensive heart failure subjects than controls (0.91 ± 0.33 vs 0.28 ± 0.16, *p* < 0.0001).

**Table 1:** Clinical Characteristics Of Study Participants

*Variable*	*Heart failure patients (n =55)*	*Controls (n = 30)*	*p*
Mean age (years)	57.8 ± 14.2	56.4 ± 18.6	0.571
Gender – female, *n* (%)	26 (47.3)	19 (47.5)	0.889
Mean SBP (mmHg)	125.2 ± 18.8	119.8 ± 13.4	0.681
Mean DBP (mmHg)	84.2 ± 12.7	74.6 ± 10.4	0.04*
Mean BMI (kg/m^2^)	27.8 ± 11.4	23.2 ± 2.1	0.03*
Mean PP (mmHg)	58.8 ± 18.5	53.0 ± 16.7	0.05
LVDD (mm)	60.9 ± 9.6	45.0 ± 7.5	0.017*
LVSD (mm)	43.7 ± 10.9	31.5 ± 7.3	0.029*
EF (%)	48.5 ± 25.9	70.6 ± 12.2	0.015*
FS (%)	22.5 ± 11.4	38.7 ± 8.1	0.035*
IVSd (mm)	13.6 ± 3.4	11.2 ± 2.6	0.024*
PWTd (mm)	12.1 ± 2.5	10.4 ± 2.1	0.021*
LAD (mm)	43.3 ± 10.5	32.2 ± 7.1	0.038*
DT (ms)	204.1 ± 61.3	172.5 ± 38.5	0.021*
IVRT (ms)	96.8 ± 32.7	79.7 ± 16.5	0.031*
IVCT (ms)	112.6 ± 39.5	82.5 ± 27.2	0.023*
Mean Tei index	0.91 ± 0.33	0.28 ± 0.16	0.001**

**Statistically significant. SBP: systolic blood pressure, DBP: diastolic blood pressure, PP: pulse pressure, BMI: body mass index, LVDD: left ventricular internal diastolic dimension, LVSD: left ventricular internal systolic dimension, EF: ejection fraction, FS: fractional shortening, IVSd: interventricular septal dimension, PWTd: posterior wall thickness, LAD: left atrial dimension, DT: deceleration time, IVRT: isovolumic relaxation time, IVCT: isovolumic contraction time.

Table 2 shows the echocardiographic parameters of the subjects with hypertensive heart failure, categorised by ejection fraction, according to the degree of systolic dysfunction. Left ventricular internal diastolic dimension, left ventricular internal systolic dimension, ejection fraction, fractional shortening and left atrial dimension were significantly different among the groups. The Tei index increased significantly as the degree of systolic dysfunction worsened in the study participants.

**Table 2:** Echocardiographic Parameters Of Study Participants According To The Severity Of Systolic Dysfunction

*Variable*	*Normal EF (n = 12)*	*Mild HF (EF 45–55%) (n = 11)*	*Moderate HF (EF 35-45%) (n = 19)*	*Severe HF (EF < 35%) (n = 13)*	*p*
Age (years)	59.1 ± 13.8	56.7 ± 10.7	56.3 ± 11.3	55.1 ± 19.0	0.887
LVDD (mm)	45.5 ± 12.3	53.9 ± 6.8	59.7 ± 8.9	63.2 ± 9.7	< 0.001 **
LVSD (mm)	27.6 ± 11.0	35.2 ± 7.5	41.5 ± 9.5	53.2 ± 8.9	< 0.001 **
EF (%)	71.8 ± 9.7	49.2 ± 8.7	36.6 ± 9.5	24.2 ± 6.5	< 0.001 **
FS (%)	36.9 ± 9.2	20.4 ± 4.3	14.4 ± 3.8	7.1 ± 3.7	< 0.001**
LAD (mm)	36.8 ± 10.0	43.6 ± 9.8	50.1 ± 7.9	40.1 ± 10.9	< 0.001**
MEARAT	1.1 ± 0.9	1.6 ± 0.6	1.4 ± 0.61	1.6 ± 1.5	0.278
DT (msec)	174.2 ± 72.6	155.2 ± 53.8	139.8 ± 65.5	154.4 ± 59.4	0.917
IVRT (ms)	112.0 ± 23.5	84.0 ± 36.0	89.6 ± 25.5	85.7 ± 40.6	0.104
IVCT (ms)	99.7 ± 31.3	121.6 ± 30.4	110.4 ± 24.7	126.2 ± 24.9	0.307
BMI (kg/m^2^)	27.7 ± 14.8	25.0 ± 5.0	24.2 ± 6.0	25.0 ± 8.5	0.622
Tei index	0.69 ± 0.12	0.82 ± 0.23	0.98 ± 0.29	1.30 ± 0.34	< 0.001 **

**Statistically significant. HF: heart failure, LVDD: left ventricular internal diastolic dimension, LVSD: left ventricular internal systolic dimension, EF: ejection fraction, FS: fractional shortening, IVSd: interventricular septal dimension in diastole, PWTd: posterior wall thickness in diastole, LAD: left atrial dimension, MEARAT: mitral E/A ratio, DT: deceleration time, IVRT: isovolumic relaxation time, TEARAT: tricuspid E/A ratio, BMI: body mass index, HF: heart failure.

Table 3 shows the linear correlation of the echocardiographic variables and echo-derived indices of systolic and diastolic function with the Tei index. Ejection fraction and fractional shortening were well correlated with the Tei index and these were statistically significant. Echocardiographic parameters of diastolic function, such as the mitral E/A ratio and deceleration time were also shown to be significantly correlated with the Tei index.

**Table 3:** Correlation Of Echocardiographic Parameters With The Derived Tei Index In The Study Population

*Variable*	*Correlation* (r)	p
LVDD	0.414	0.002 **
LVSD	0.596	< 0.001 **
EF	–0.697	< 0.001 **
FS	–0.580	0.001 **
LAD	0.155	0.267
MEARAT	0.246	0.030 *
DT	0.219	0.032 *

*Statistically significant. LVDD: left ventricular internal diastolic dimension, LVSD: left ventricular internal systolic dimension, EF: ejection fraction, FS: fractional shortening, LAD: left atrial dimension, MEARAT: mitral E/A ratio, DT: deceleration time.

## Discussion

Heart failure is a major and growing public health concern globally. The aetiologies of heart failure in Africans include hypertension, cardiomyopathies and rheumatic heart disease, as reported by Ntusi *et al.* and Amoah *et al.*, with hypertension remaining the commonest cause.^16^,^17^

This study shows that the Tei index of myocardial performance is significantly different between patients with hypertensive heart failure and normotensive subjects. It also shows that the higher the degree of systolic dysfunction, the higher the Tei index. Another important finding was that the Tei index correlated significantly with other conventional indices of systolic and/or diastolic dysfunction among Africans with hypertensive heart failure. It may therefore be a clinically useful index of overall dysfunction among black Africans with heart failure, in a similar way that ejection fraction, fractional shortening, deceleration time and isovolumic relaxation time are useful for risk estimation, treatment evaluation and prognosis in subjects with heart failure.

Traditionally, assessment of left ventricular function has focused on measurement of ejection fraction and diastolic indices using Doppler measurements for risk stratification and treatment evaluation.^18^ These measurements provide important prognostic information regarding clinical outcome in patients with heart failure. However, assessment of left ventricular diastolic dysfunction may be more challenging because diastolic function is more difficult to estimate and varies with age and loading conditions.^19^ Heart failure is usually associated with both systolic and diastolic dysfunction and a combined index of overall risk estimation may be more useful in risk stratification, treatment evaluation and prognosis.

The Tei index is a function of cardiac intervals derived from ejection time, contractility and relaxation period. It therefore indicates combined systolic and diastolic dysfunction and appears to be a more ideal test for overall dysfunction in heart failure. It has been shown to be independent of ventricular loading conditions, is easily reproducible, and assesses the overall function of the heart.^20^ The Tei index has been shown to correlate with combined systolic and diastolic dysfunction in several heart diseases, including dilated cardiomyopathy and amyloidosis.^10^-^12^ Systolic time intervals have been shown to correlate with systolic left ventricular performance.^21^,^22^ Similarly, diastolic time intervals have been shown to correlate with left ventricular diastolic performance in several patient populations.^23^,^24^

The Tei index is non-invasive and easily obtainable. It has also been shown to have prognostic significance in subjects with heart failure.^8^,^9^,^12^,^15^,^25^ Ejection fraction is the most commonly used method to estimate left ventricular function and was well correlated with the Tei index in our study. The Tei index was significantly higher among subjects with higher systolic dysfunction. Fractional shortening, deceleration time and mitral E/A ratio also correlated well with the Tei index. Our study is in agreement with similar studies that had shown that the Tei index can be a useful prognostic index and an accurate estimate of overall left ventricular function in a wide variety of subjects.^8^,^9^,^12^,^26^,^27^

Several authors have argued that some degree of systolic and diastolic dysfunction coexist in almost all patient with heart failure.^28^,^29^ A measurement such as the Tei index therefore seems to be more appropriate for estimating overall ventricular function. This has also been reported by other authors.^9^,^15^ The Tei index provides useful information and clinical value in patients with heart failure of various origins, including hypertensive heart disease, rheumatic heart disease, dilated cardiomyopathy and alcoholic heart muscle disease. The prognostic usefulness of the Tei index as a combined index of overall heart function in black African subjects should be further studied.

As promising as the Tei index is, it is not yet clear whether it has any role in the aetiological classification of heart failure or in differentiating systolic from diastolic heart failure. These could be assessed by further prospective studies.

## Conclusion

The Tei index correlated well with conventional indices of systolic and diastolic dysfunction in subjects with hypertensive heart failure. It may be an additional tool for risk stratification, treatment evaluation and prognosis in black Africans.
